# Absence of the Common Gamma Chain (γ_c_), a Critical Component of the Type I IL-4 Receptor, Increases the Severity of Allergic Lung Inflammation

**DOI:** 10.1371/journal.pone.0071344

**Published:** 2013-08-05

**Authors:** Preeta Dasgupta, Xiulan Qi, Elizabeth P. Smith, Achsah D. Keegan

**Affiliations:** 1 Department of Microbiology and Immunology, Center for Vascular and Inflammatory Diseases, University of Maryland School of Medicine, Baltimore, Maryland, United States of America; 2 Center for Vascular and Inflammatory Diseases, University of Maryland School of Medicine, Baltimore, Maryland, United States of America; 3 Center for Vascular and Inflammatory Diseases, University of Maryland School of Medicine, Baltimore, Maryland, United States of America; 4 Department of Microbiology and Immunology, Center for Vascular and Inflammatory Diseases, University of Maryland School of Medicine, Baltimore, Maryland, United States of America; University of Tübingen, Germany

## Abstract

The T_H_2 cytokines, IL-4 and IL-13, play critical roles in inducing allergic lung inflammation and drive the alternative activation of macrophages (AAM). Although both cytokines share receptor subunits, IL-4 and IL-13 have differential roles in asthma pathogenesis: IL-4 regulates T_H_2 cell differentiation, while IL-13 regulates airway hyperreactivity and mucus production. Aside from controlling T_H_2 differentiation, the unique contribution of IL-4 signaling via the Type I receptor in airway inflammation remains unclear. Therefore, we analyzed responses in mice deficient in gamma c (γ_c_) to elucidate the role of the Type I IL-4 receptor. OVA primed CD4^+^ OT-II T cells were adoptively transferred into RAG2^−/−^ and γ_c_^−/−^ mice and allergic lung disease was induced. Both γ_c_^−/−^ and γ_c_xRAG2^−/−^ mice developed increased pulmonary inflammation and eosinophilia upon OVA challenge, compared to RAG2^−/−^ mice. Characteristic AAM proteins FIZZ1 and YM1 were expressed in lung epithelial cells in both mouse strains, but greater numbers of FIZZ1+ or YM1+ airways were present in γ_c_^−/−^ mice. Absence of γ_c_ in macrophages, however, resulted in reduced YM1 expression. We observed higher T_H_2 cytokine levels in the BAL and an altered DC phenotype in the γ_c_^−/−^ recipient mice suggesting the potential for dysregulated T cell and dendritic cell (DC) activation in the γ_c-_deficient environment. These results demonstrate that in absence of the Type I IL-4R, the Type II R can mediate allergic responses in the presence of T_H_2 effectors. However, the Type I R regulates AAM protein expression in macrophages.

## Introduction

IL-4 and IL-13 are central mediators of asthmatic responses. They initiate and propagate hallmark features of asthma such as pulmonary inflammation, eosinophilia, mucus hypersecretion and airway hyperreactivity by engaging shared receptor complexes and signaling proteins [1], [2], [3], [4]. IL-4 alone, binds to the Type I receptor (R), composed of the IL-4Rα chain and IL-2Rγ (common gamma (γ_c_)) chain (reviewed in [5]). Both IL-4 and IL-13, however, can signal through the Type II R (composed of IL-4Rα and IL-13Rα1) [5].

While both IL-4 and IL-13 can elicit asthma pathology when provided exogenously, it is evident that they mediate different responses *in vivo* [2], [3], [6], [7]. IL-4 is critical for T_H_2 cell differentiation and IgE synthesis, while IL-13 is predominantly responsible for inducing airway hyperesponsiveness and mucus secretion. The reason for this separation of duties is not well understood; relative abundance and differential usage of receptor complexes and signaling pathways in different cell types, together with greater quantities of IL-13 (than IL-4) produced during T_H_2 responses have been proposed to explain these observations (reviewed in [8]). A recent publication also suggested that there may be distinct cellular expression and localization of IL-4 and IL-13 [9].

The relative contributions of the Type I R and the Type II R to asthma pathophysiology are only now being investigated. The unique contributions of the Type II receptor in allergic lung inflammation were examined using IL-13Rα1^−/−^ mice. It was reported that mucus secretion, airway resistance, eotaxin production and induction of pro-fibrotic mediators such as TGFβ were completely dependent on the IL-13Rα1 chain, and thus the Type II receptor [10], [11]. However, T_H_2 cell differentiation, IgE secretion, and recruitment of eosinophils into the lungs could occur independently of IL-13Rα1.

IL-4 and IL-13 also stimulate alternative activation of macrophages (AAM). AAM express a distinctive set of proteins such as Arginase 1 (Arg1), found in inflammatory zone (FIZZ)-1-4 and some members of the chitinase family such as acidic mammalian chitinase (AMCase) and YM1/2. DNA microarray analysis of cells isolated from allergen or IL-4 treated WT or IL-13Rα1^−/−^ mice revealed that several AAM genes were differentially regulated by the Type I and Type II R. Munitz *et.al.* showed that allergen- and IL-4-induced FIZZ1 (*Retnla)* expression levels were similar in both WT and IL-13Rα1^−/−^ mice, but induction of chitinase (*Chia*) was completely dependent on IL-13Rα1 [10]. Our studies indicated that IL-4 induces significantly greater expression of AAM genes (FIZZ1, YM1 and Arg1) *in vitro*, when compared to IL-13 [12].

The above findings clearly demonstrated that IL-4 or IL-13 signaling through the Type II R is not required for mediating pulmonary inflammation and eosinophilia, and suggest the hypothesis that the Type I receptor is responsible for controlling the inflammatory response. Therefore, to test the specific role of the Type I R, we assessed the degree of airway inflammation, eosinophilia, and AAM stimulation upon allergen priming and challenge in mice lacking γ_c_ (and the Type I R). Here we report that γ_c_ deficient mice developed increased pulmonary inflammation and eosinophilia upon OVA challenge when compared to RAG2^−/−^ mice when provided with OVA-specific T-cells. Although significantly higher numbers of FIZZ1+ or YM1+ airways were detected in γ_c_^−/−^ mice, absence of the Type I R in macrophages caused reduced YM1 expression in these cells. These results suggest that the Type I and Type II receptors have redundant functions *in vivo* and the Type II R can mediate effector allergic responses in absence of the Type I R. However, in macrophages the Type I R regulates YM1 protein expression.

## Materials and Methods

### Ethics Statement

All experimental procedures on mice were performed in accordance to guidelines issued by the National Institutes of Health Guide for the Care and Use of Laboratory Animals and approved by the Institutional Animal Care and Use Committee at UMB.

### Mice

γ_c_^−/−^ mice and OT-II transgenic mice on a C57BL/6 background were acquired from Jackson Labs and bred in the animal care facility at the University of Maryland, Baltimore (UMB). Mice deficient in RAG2 (B6.RAG2^−/−^) were purchased from Taconic (Germantown, NY). γ_c_xRAG2^−/−^ mice were obtained from Dr. Paul Antony at UMB and bred in house.

### Adoptive Transfer of in vivo Primed CD4 T Cells

OT-II transgenic mice were immunized with 100 µg of chicken egg ovalbumin (OVA; Sigma-Aldrich, St. Louis, MO) adsorbed to aluminum hydroxide (alum; Sigma-Aldrich) intraperitoneally (i.p) and LNs and spleens were harvested 10 days later. CD4+ T cells present in these tissues were purified by negative selection (Easy Sep kit, Stem Cell Technologies, Vancouver, Canada). Following this, i*n vivo* primed CD4+ T cells were injected intravenously (i.v.) via the tail vein in recipient mice (5×10^6^ cells/mouse).

### Antigen Sensitization and Challenge

Mice were sensitized and challenged with OVA using a protocol described earlier [13]. Briefly, mice were immunized with either 100 µg of OVA/alum or alum alone on day 1 and day 6. After the last sensitization step, mice were challenged with aerosolized 1% OVA in PBS for 40 minutes each day on days 12 and 14.

### Evaluation of Airway Inflammation

Bronchial lavage was performed 48 hours after the last OVA challenge as described previously [13]. The cellular component of the bronchoalveolar lavage (BAL) was used to determine total and differential cell counts and the supernatant was used for cytokine analysis.

### Lung Histology and Immunohistochemistry

Lung histology sections were prepared as described [13]. Briefly, mouse lungs were perfused with 10–15 ml of PBS followed by fixation with 10% formalin. The tissues were then processed, embedded in paraffin and sectioned. After deparaffinization, slides were stained with Hematoxylin and Eosin (H&E) or Periodic acid Schiff (PAS). For immunohistochemistry, deparaffinized sections were incubated with 10% goat serum and stained with a 1∶100 dilution of rabbit anti-mouse FIZZ1 (Abcam, Cambridge, MA) or 1∶100 dilution of rabbit anti-mouse YM1 (Stem Cell Technologies, Vancouver, Canada). The histology sections were prepared by E.S., who generated unique slide numbers. The sections were then evaluated by P.D. without knowledge of the identity of the experimental groups. Areas of the slide that were representative for the whole group were photographed and digitally processed using CoolSnap (Roper Scientific, Trenton, NJ). For cell counts, photomicrographs of 10 100×fields were taken per mouse and average number of cells per high power field were calculated and graphed.

### Assessment of Airway Remodeling

Lung sections were stained with Masson’s Trichrome to detect collagen deposition. The collagen content around the airways was quantified using NIH Image J software (National Institutes of Health, Bethesda, MD) [13]. Airway smooth muscle thickness was measured using H&E stained lung sections as described previously [13].

### NK Cell Depletion

Anti-asialo GM1 antibody was obtained from Wako Chemicals USA. Mice were injected intraperitoneally with 30 µg anti-asialo GM1 antibody in a volume of 300 µl, starting on day -2 and every 5 days thereafter over the course of the study.

### NK Cell Isolation and Transfer

Splenocytes from WT C57BL/6 mice or B6.STAT6^−/−^ mice were enriched for NK cells using the Stem Cell Technolgies NK cell enrichment kit. WT or STAT6^−/−^ NK cells (1×10^6^) were transferred into γ_c_xRAG2^−/−^ mice through the tail vein at the time CD4^+^ T cell adoptive transfer.

### Preparation of Lung Digests

Lung tissue samples were harvested from mice 48 hours after the last challenge. The tissue was minced into small pieces and incubated with serum-free RPMI medium containing 150 U/ml Collagenase Type IV (Worthington Biochemicals) and 10 U/ml DNase (Roche) for 1 hour at 37°C. Cells were spun down and RBC lysis performed. After washing, cells were resuspended in complete medium and counted before use.

### Cytokine and Chemokine Analysis

Cytokines in the BAL fluid or cell culture supernatants were analyzed by using individual ELISA kits for IL-4 (Pierce Thermo Scientific, Rockford, IL; BioLegend, San Diego, CA), IL-5, IL-13 and IFNγ (all from R&D Systems, Minneapolis, MN).

### FACS Analysis

Single cell suspensions of splenocytes or BAL cells were incubated with Fc Block (2.4G2, BD Biosciences) followed by staining with fluorochrome-conjugated antibodies to surface markers (from BD Biosciences: CD4-PE, CD4-Alexa Fluor 647, CD11b-PE, OX40L-PE, CD11c-FITC, F4/80-Alexa Fluor 647 and CD69-PerCP-Cy5.5. From eBioscience: CD44-PerCP-Cy5.5 and CD62L-PerCP-Cy5.5). Cells were washed twice with FACS buffer and analyzed directly or after fixing with 4% paraformaldehyde by using a FACS Calibur machine (Becton Dickinson, Franklin Lakes, NJ). For measurement of intracellular cytokine staining, cells were fixed and permeabilized using a BD Cytofix/Cytoperm kit (BD Biosciences), followed by staining with antibodies for intracellular proteins. After incubation with antibodies, cells were washed twice with Perm Wash Buffer and resuspended in FACS buffer before acquisition. Data was analyzed by using FlowJo software (Treestar, CostaMesa, CA).

### Statistical Analysis

Anova single factor data analysis tool was used to compare the differences between two groups and to calculate significance values. p values of ≤0.05 were considered statistically significant.

## Results

To determine the contribution of the Type I R in inducing features of allergic lung disease, we utilized γ_c_^−/−^ mice. Since T cells play a critical role in initiating and propagating asthma and γ_c_^−/−^ mice lack T-cells, we used an asthma model wherein we provided *in vivo*-primed OVA-specific OT-II T cells to γ_c_–sufficient RAG2^−/−^, γ_c_^−/−^, or γ_c_xRAG2^−/−^ mice using a previously established transfer model [13]. These T cells expressed γ_c_ and could respond to IL-2, IL-4, and IL-7, cytokines required for T_H_2 differentiation and T cell survival respectively. To demonstrate activation of the *in vivo*-primed T cells, splenocytes isolated from either unimmunized or OVA/alum-immunized OT-II mice were cultured *in vitro* for 48 hours in the presence or absence of anti-CD3 and anti-CD28; expression of cell surface activation markers (CD44, CD62L and CD69) on OVA-specific CD4+ T cells was monitored by flow cytometry. When cells were isolated from unimmunized mice and cultured in media only, the majority of the cells were CD44^lo^ and CD62L^hi^ (Supporting Information, Figure S1A). Upon OVA/alum immunization, the percentage of CD44^hi^ cells increased. Cells that were isolated from OVA/alum-immunized mice and stimulated *in vitro* showed the maximum upregulation of CD44 expression. In addition, CD62L expression was reduced and nearly 80% of the cells were CD69^+^. This showed that OVA/alum priming in OT-II mice induced T cell activation. Similar to our findings in DO11.10 mice [13], immunization of OT-II mice with OVA/alum induced greater IL-4 and IL-5 production by primed cells in comparison to naïve cells (Supporting Information, Figure S1B). In contrast, IFNγ levels between the two groups were similar. These data suggest that the OVA-specific T cells were skewed to a T_H_2 phenotype.

### Effect of γ_c_ Deficiency on Allergic Lung Inflammation

**Figure 1:**
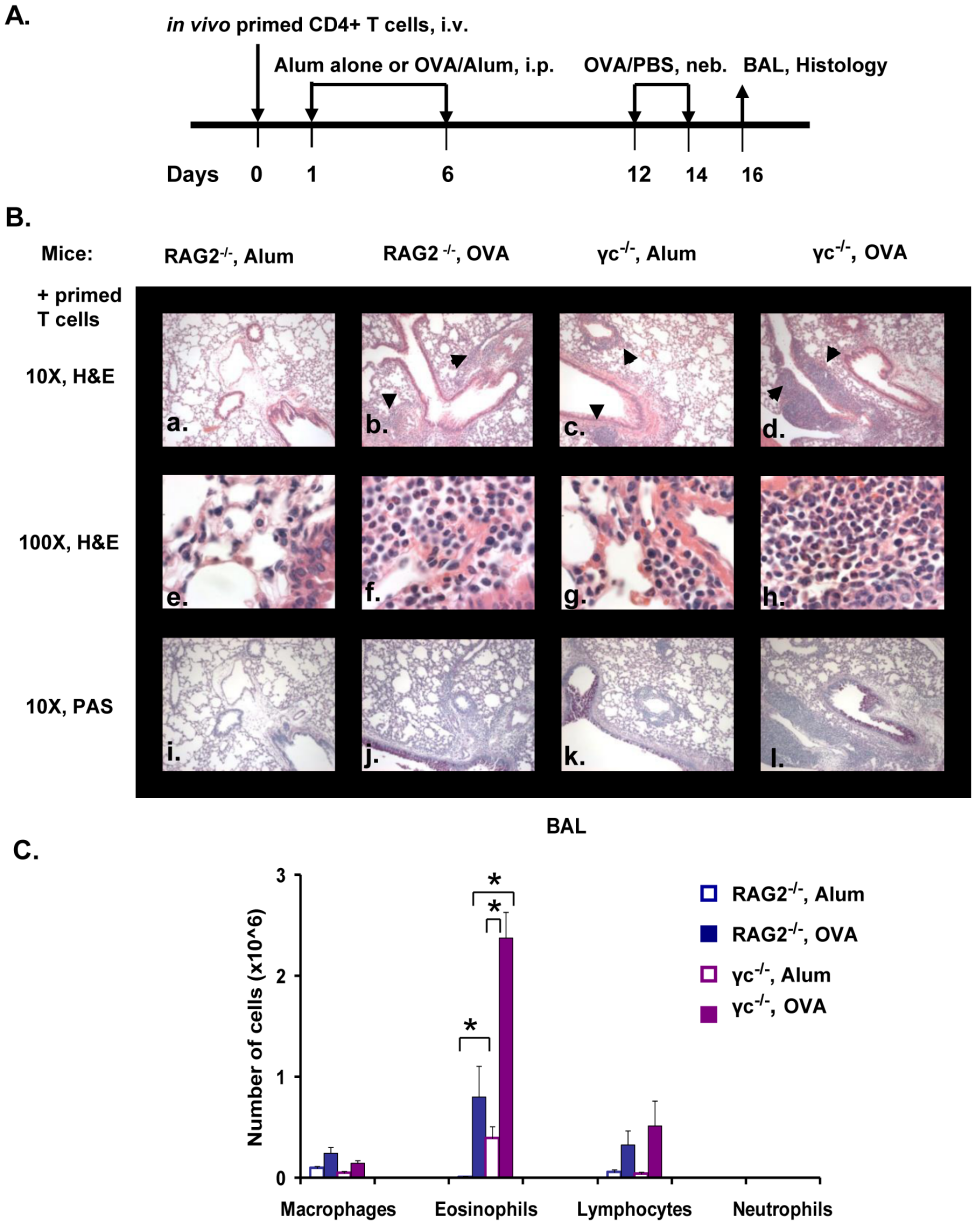
Degree of eosinophilia and inflammation in RAG2^−/−^ or γ_c_^−/−^ mice. The asthma protocol used in this study is depicted in (A). (B) H&E (panels a-d- 10X; panels e-h- 100X) and PAS (panels i-l- 10X) stained lung sections of B6.RAG2^−/−^ and B6.γ_c_^−/−^ mice. Arrows point areas of inflammation. (C) The number of macrophages, eosinophils, lymphocytes and neutrophils present in the BAL are shown here. *p<0.05. n = 5 each for RAG2^−/−^ or γ_c_^−/−^ mice treated with OVA/alum, n = 3 for alum treated mice. Representative data from one of three experiments is shown.

To delineate the role of the γ_c_ chain and the Type I R in allergic airway disease, we induced allergic inflammation in γ_c_^−/−^ mice. Since these mice lack endogenous lymphocytes, RAG2^−/−^ mice were used as controls. *In vivo*-primed CD4+ T cells isolated from OT-II transgenic mice were adoptively transferred into RAG2^−/−^ and γ_c_^−/−^ mice and allergic lung inflammation was induced (Figure 1A). Lung histology analysis revealed that RAG2^−/−^ mice developed no inflammation or epithelial cell-induced mucus production in the absence of OVA priming (Figure 1B panels a, e & i). Immunization of RAG2^−/−^ mice with OVA, on the other hand caused enhanced peribronchial and perivascular inflammation (Figure 1B panel b), recruitment of eosinophils into the lung (panel f) and mucus production (panel j). Surprisingly, we found that absence of the γ_c_ chain in recipient mice led to increased lung inflammation and eosinophilia after OVA challenge even in the absence of OVA priming (Figure 1B panels c & g). In contrast to the RAG2^−/−^ alum group (panel i), the epithelial cells in this group were PAS^+^ (panel k). Finally, when γ_c_^−/−^ mice were primed and challenged with OVA there was massive influx of cells into the lungs (Figure 1B panel d); most of these cells were identified as eosinophils (panel h). There was mucus hypersecretion in these mice as well (panel l). Differential counts of BAL cells further demonstrated that OVA priming and challenge led to a significant increase in the number of eosiniophils in γ_c_^−/−^ mice when compared to RAG2^−/−^ mice (Figure 1C).

**Figure 2:**
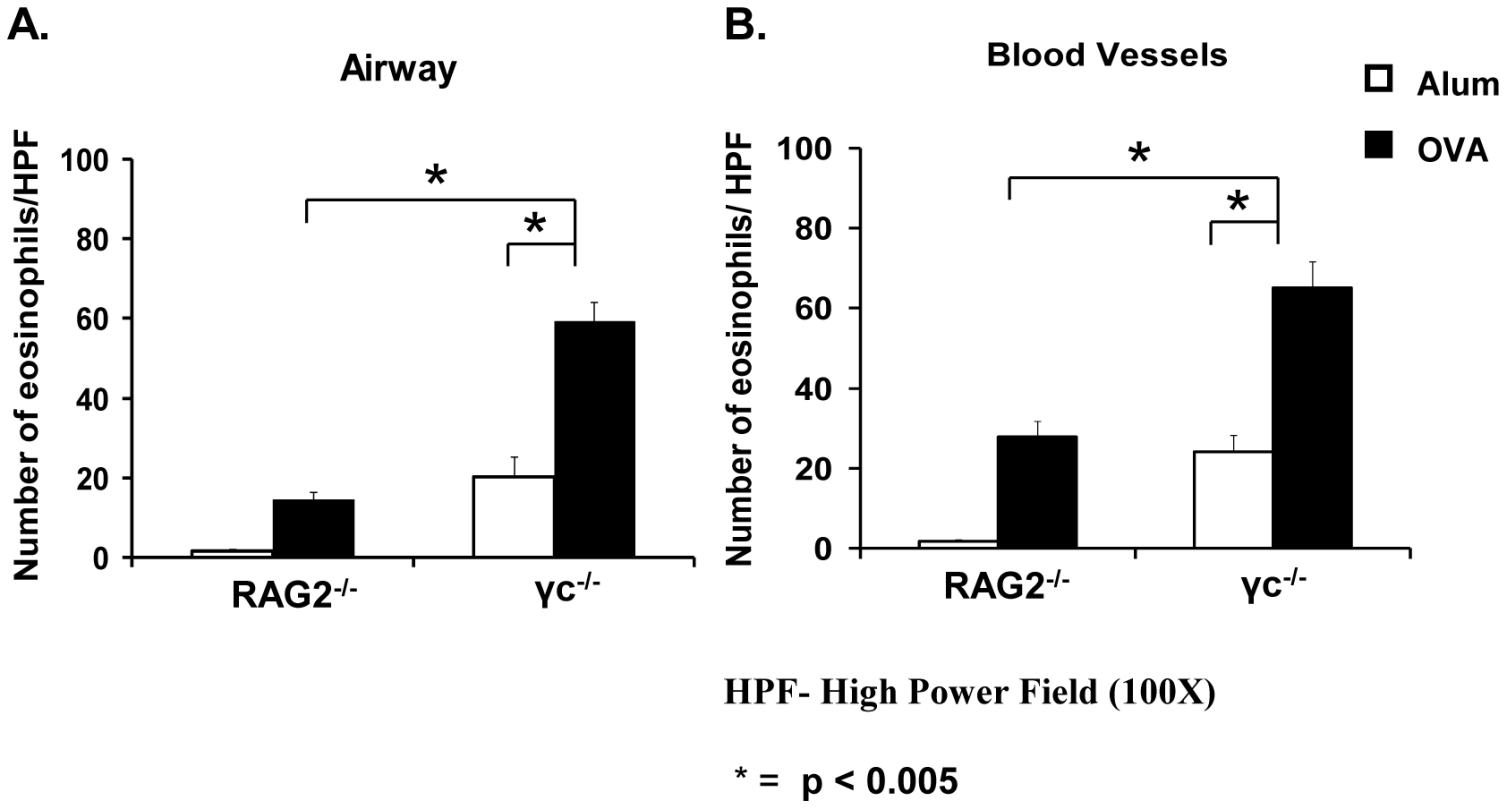
Absence of γ_c_ causes enhanced eosinophil accumulation in the lung. Lung sections of Alum- or OVA/alum-primed and OVA-challenged mice mentioned in Fig.1 were stained with H&E. Photomicrographs of five high power fields (HPF; 100X) of cells around the airways and blood vessels (total of 10 HPF) were used to count the number of eosinophils. Eosinophils in each lung section was counted and graphed. Number of cells around the airways (A) and blood vessels (B) are shown. Open bars represent alum-primed mice; closed bars represent OVA-primed mice. Data represented as cell counts ± SEM. *p<0.005. n = 5 for OVA-treated mice, n = 3 for alum-treated. Representative data from one of three experiments is shown.

The differences in eosinophil counts in the BAL (Figure 1C) in the two mouse strains were recapitulated in the lung tissue. The number of eosinophils recruited to the airways and blood vessels in both alum- and OVA-primed and OVA-challenged γ_c_^−/−^ mice were significantly increased in comparison to their RAG2^−/−^ counterparts (Figure 2).

**Figure 3:**
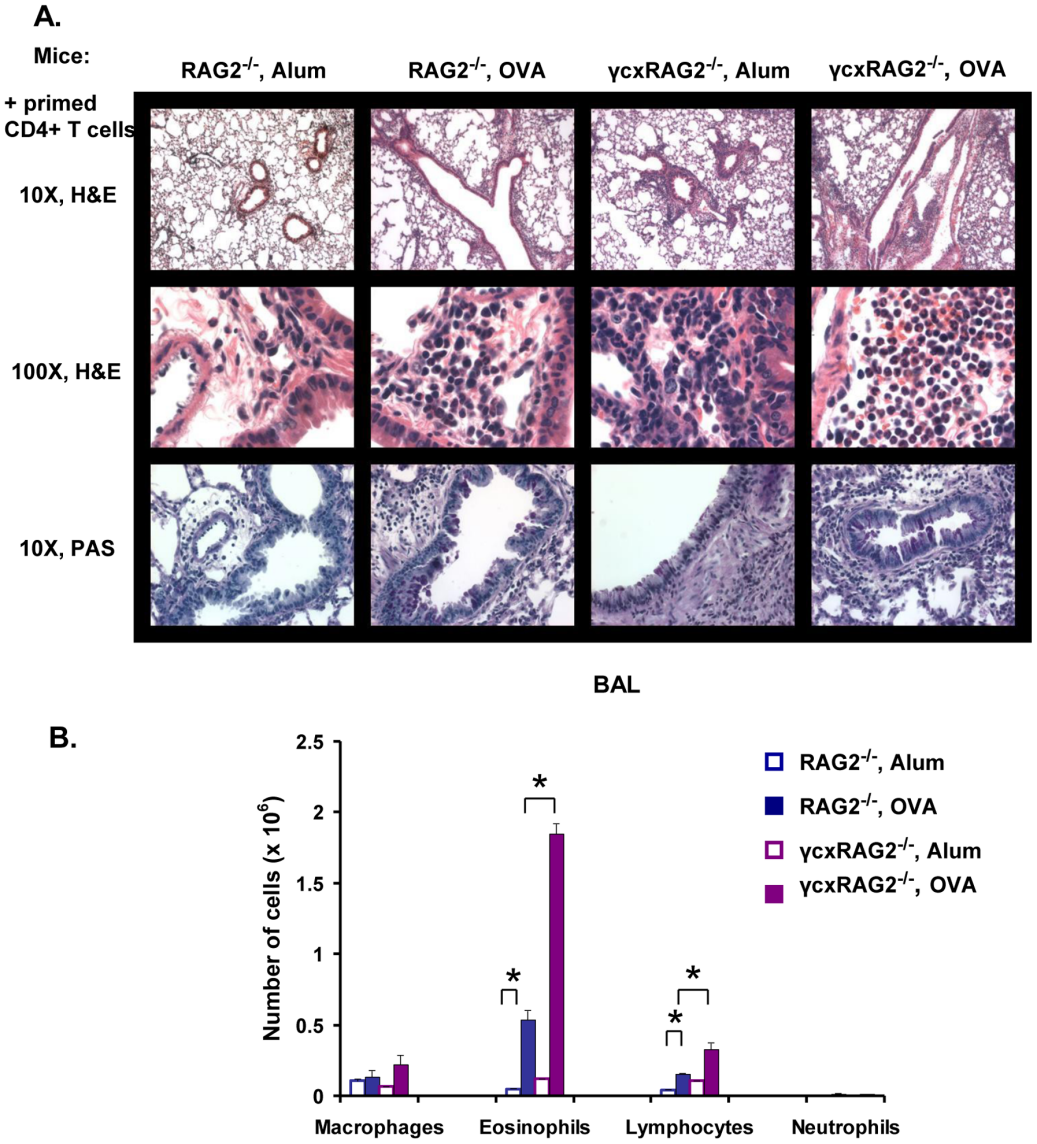
γcxRAG2^−/−^ mice also develop enhanced allergic lung inflammation. (A) H&E (10X and 100X) and PAS (10X) stained lung sections of RAG2^−/−^ and γcxRAG2^−/−^ mice are shown here. (B) The number of macrophages, eosinophils, lymphocytes and neutrophils present in the BAL in these mice are represented in the form of bar graphs. *p<0.05. n = 5 for OVA-primed mice, n = 3 for alum-primed. Representative data from one of two experiments is shown.

To rule out the possibility that small differences in the genetic background of these mice were causing the differences in allergic lung inflammation seen in these mice, we repeated the above experiment with RAG2^−/−^ mice and γ_c_xRAG2^−/−^ mice. γ_c_ deficient mice on a RAG2^−/−^ background still developed significantly higher pulmonary inflammation and eosinophilia (Fig. 3A and 3B). Thus, the responses seen in γ_c_^−/−^ and γ_c_xRAG2^−/−^ mice were essentially the same in all aspects and these mice were used interchangeably. These results show that the Type I R is not required for inflammatory response. They further suggest that in the absence of γ_c_ and the Type I R, the Type II R can mediate the pulmonary inflammatory response as well as mucus production. Moreover, deficiency of γ_c_ in cells other than T cells led to exaggerated asthma pathology.

### Effect of γ_c_ Deficiency on FIZZ1 and YM1 Protein Expression

Gene expression profiling of lungs from allergen challenged IL-13Rα1 deficient mice had identified FIZZ1 upregulation as being completely independent of the Type II R while YM1 was only partially dependent [10]. This suggested that the Type I R may contribute to AAM gene expression. We have previously found that bone marrow-derived macrophages (BMM) isolated from WT mice induced robust induction of FIZZ1 and YM1 transcripts when stimulated with IL-4 *in vitro* [12]. In contrast, IL-4 treated BMM from γ_c_^−/−^ mice demonstrated significantly reduced AAM gene expression. Thus, we examined if expression of FIZZ1 and YM1 protein was reduced *in vivo* during allergic lung inflammation when γ_c_ and the Type I R was absent.

**Figure 4:**
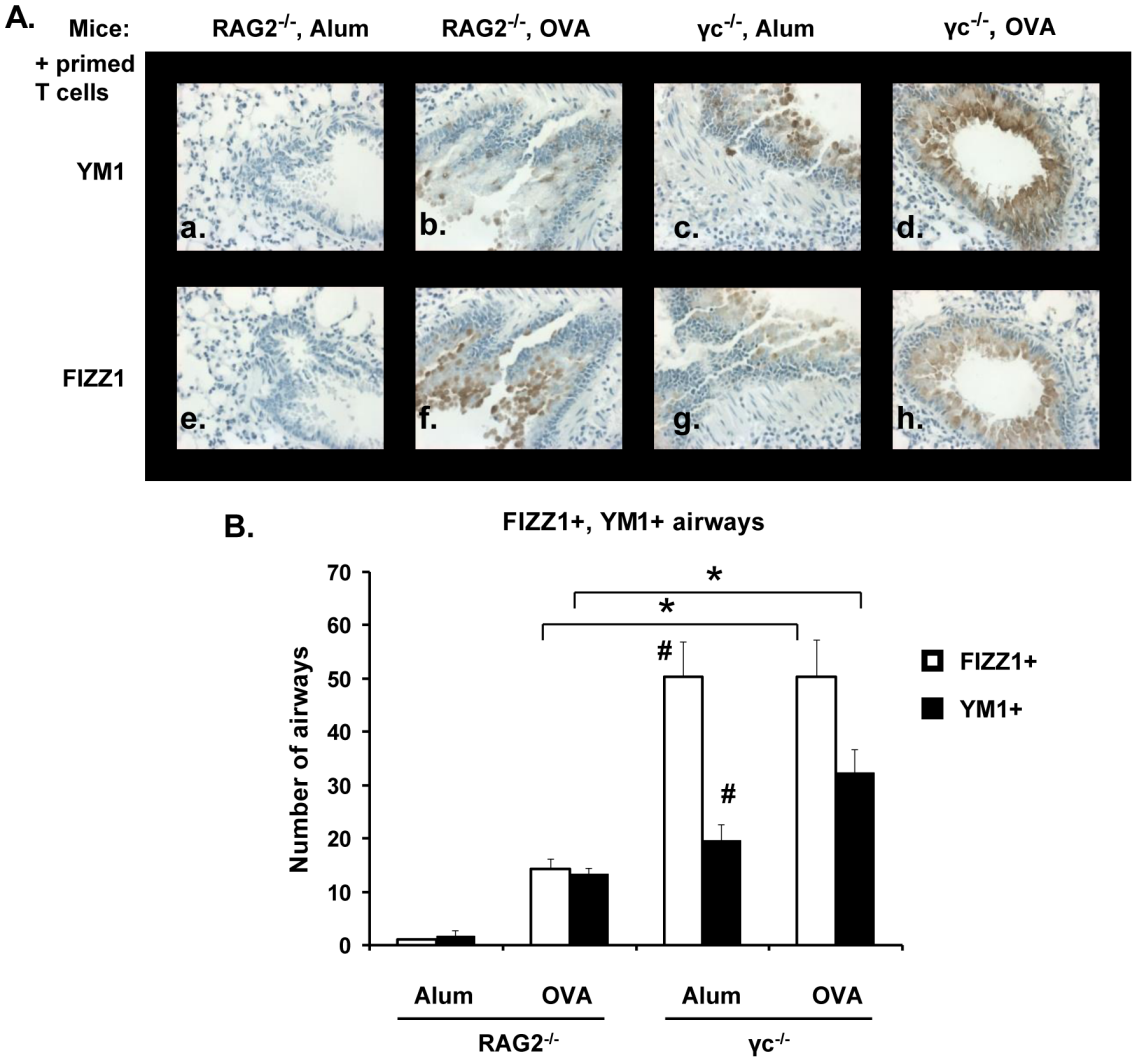
FIZZ1 and YM1 expression in lung epithelial cells. Allergic lung inflammation was induced in RAG2^−/−^and γ_c_^−/−^ mice as mentioned in Fig. 1. FIZZ1 and YM1 expression was analyzed in serial sections of mouse lungs by immunohistochemistry. Photomicrographs (40X magnification) of YM1 (panels a-d) and FIZZ1 (panels e-h) expression in epithelial cells in representative lung sections are shown. (B) The number of YM1+ or FIZZ1+ airways in each group of mice was counted. Data represented as number of airways ± SEM. *p<0.05, # (p<0.01) represents statistically significant differences between alum-primed RAG2^−/−^and γ_c_^−/−^ mice. n = 5 for OVA-primed mice, n = 3 for alum primed. Representative data from one of three experiments is shown.

To determine if macrophages or epithelial cells or both were producing FIZZ1 and YM1, immunohistochemical staining was performed on serial lung sections from alum- or OVA-primed and OVA-challenged RAG2^−/−^ and γ_c_^−/−^ mice. No YM1 or FIZZ1 was detected in lung epithelial cells in RAG2^−/−^ mice in the absence of OVA priming (Figure 4A, panels a & e), but expression of these proteins was increased upon OVA priming (panels b & f). However, both alum- and OVA-primed γ_c_^−/−^ epithelial cells stained strongly for YM1 and FIZZ1 after OVA challenge (panels c-d & g-h). Quantification of YM1^+^ or FIZZ1^+^ airways in both mice showed a significant increase in the numbers of airways expressing these proteins in γ_c_^−/−^ mice over RAG2^−/−^ mice (Figure 4B).

**Figure 5:**
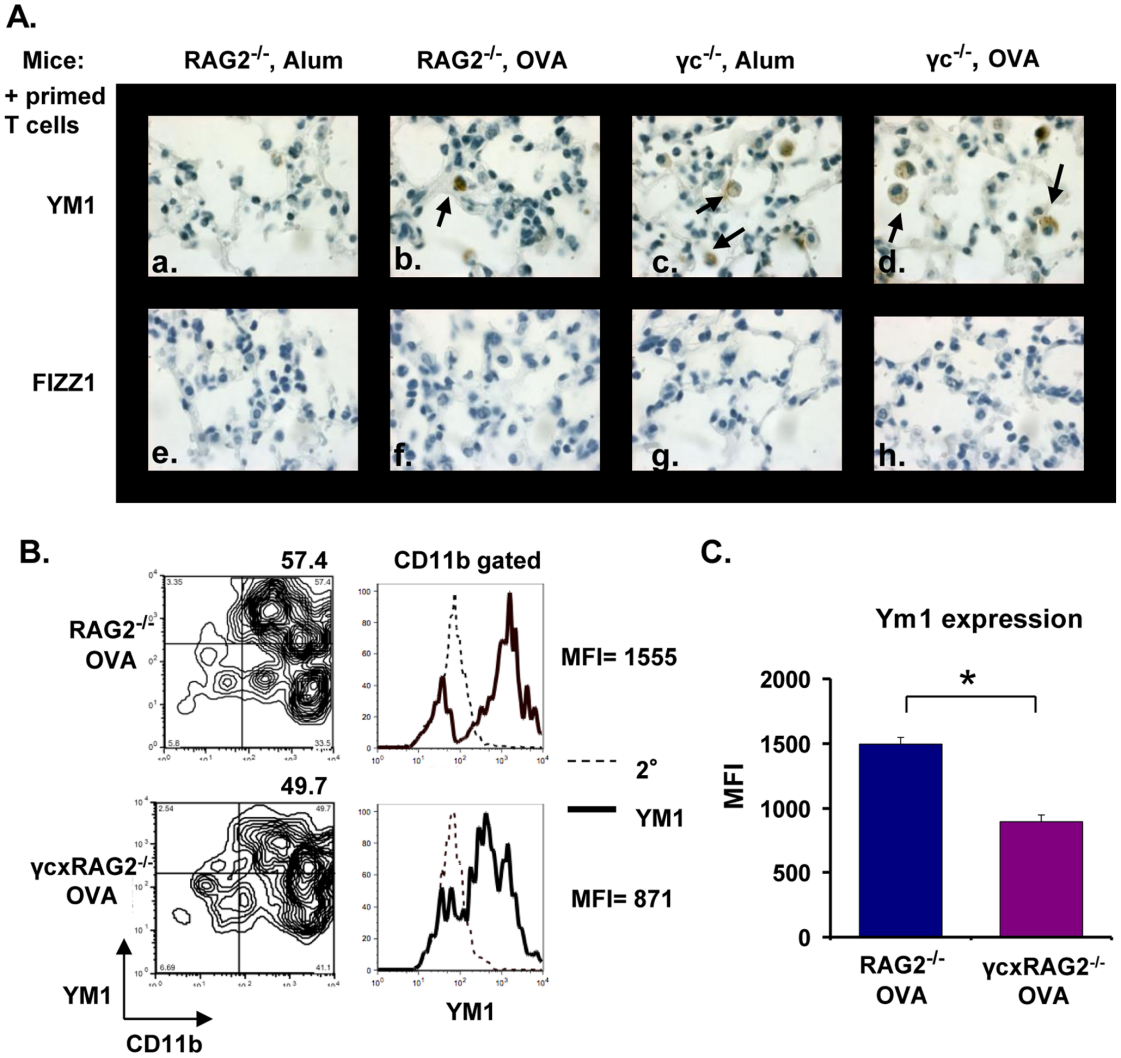
FIZZ1 and YM1 expression in macrophages. Allergic lung disease was induced in RAG2^−/−^ and γ_c_^−/−^ mice as shown in Figure 1. Serial sections of mouse lungs were stained for FIZZ1 and YM1 by immunohistochemistry. Photomicrographs (100X magnification) of YM1 (panels a-d) and FIZZ1 (panels e-h) expression in macrophages in representative lung sections are shown. YM1^+^ macrophages are indicated by arrows. (B) BAL cells from RAG2^−/−^ and γcxRAG2^−/−^ mice immunized and challenged with OVA were collected and analyzed by FACS. Cells were labeled with a fluorochrome-conjugated antibody to CD11b, stained with an antibody to YM1, followed by a secondary antibody conjugated to Alexa Fluor 647 (solid histogram). Secondary antibody staining alone was used as control (dashed histogram). Macrophages were gated based on forward by side scatter and then on CD11b expression. MFI = Mean Fluorescence Intensity of YM1 expression. (C) The average MFI of YM1 staining in macrophages from RAG2^−/−^ and γcxRAG2^−/−^ mice from different experiments is shown. *p<0.05, n = 5 for OVA-primed mice, n = 3 for alum primed. Representative data from one of two experiments is shown.

Unlike epithelial cells, which expressed both YM1 and FIZZ1 protein, macrophages expressed only YM1 (Figure 5A). In contrast to the epithelial cells, however, YM1 expression in γ_c_^−/−^ macrophages was found to be less intense than macrophages present in RAG2^−/−^ mice (panels b-d). This observation was confirmed by monitoring YM1 expression in BAL macrophages by flow cytometry. Although the percentages of CD11b^+^YM1^+^ cells were similar in both groups of mice, the mean fluorescence intensity (MFI) of YM1 staining was reduced by half when these cells lacked γ_c_ (Figure 5B). The differences in YM1 staining intensity in the two mouse strains were significant (Figure 5C).

Taken together, these results indicate that the Type I R regulates YM1 protein expression in macrophages *in vivo*. This is consistent with *in vitro* studies that have demonstrated that IL-4 induced greater YM1 gene and protein expression than IL-13 in BMM cells [12]. Epithelial cells, which express only the Type II R, can still express both YM1 and FIZZ1. Moreover, deficiency of γ_c_ led to enhanced YM1 and FIZZ1 production in these cells after OVA challenge whether or not the recipients were primed with OVA.

### Effect of γ_c_ Deficiency on Airway Remodeling

**Figure 6:**
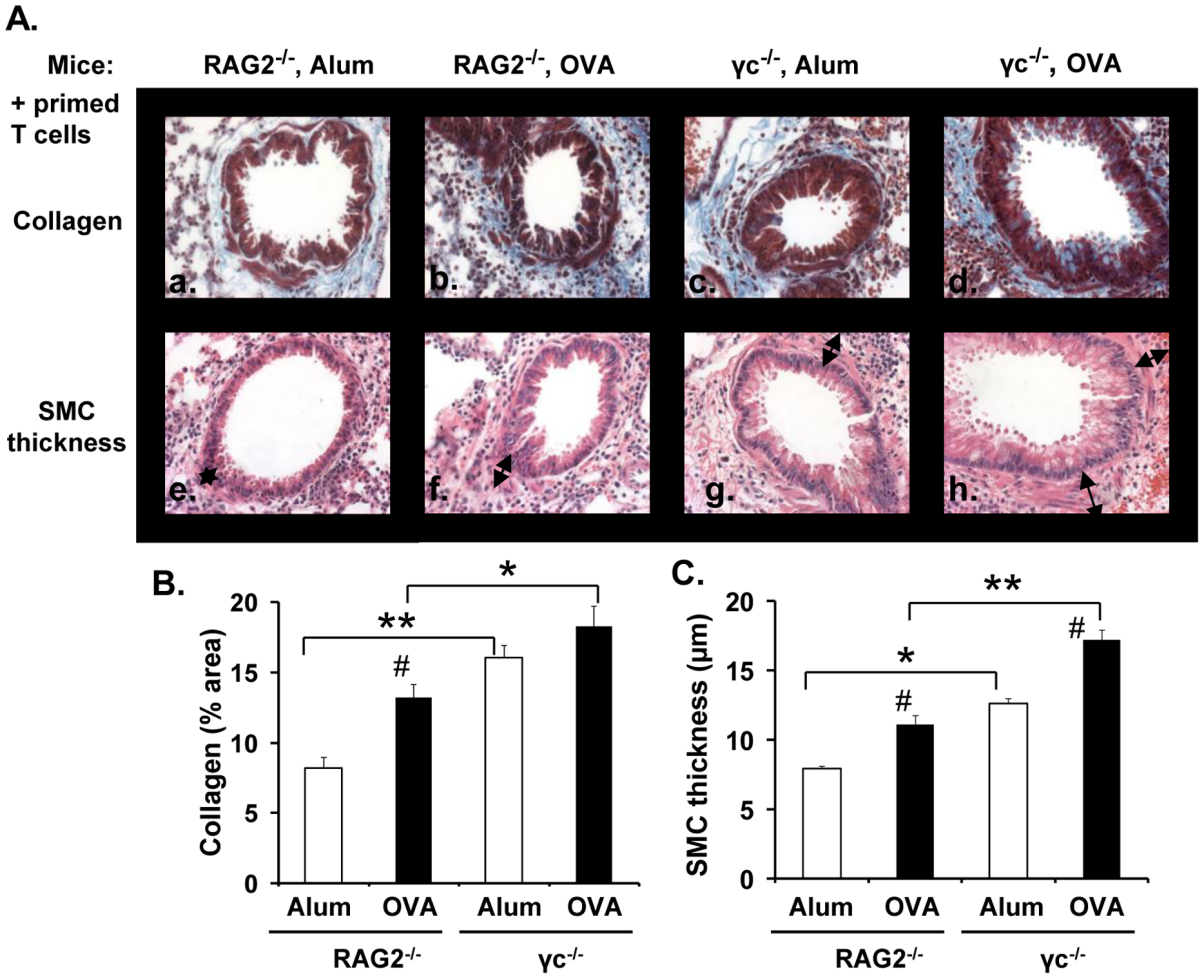
Enhanced airway remodeling in γ_c_ deficient mice. RAG2^−/−^and γ_c_^−/−^ mice were subjected to the asthma protocol as described in Figure 1. (A) Lung sections of mice were stained with Masson’s Trichrome. Photomicrographs of collagen deposition around the airways (panels a-d) in RAG2^−/−^and γ_c_^−/−^ mice primed with Alum or OVA/alum are shown. Panels e-h: photomicrographs (40X) of the airway smooth muscle (ASM) layer in H&E stained lung sections from each mouse group. Arrows depict the thickness of the ASM layer (transverse section). (B) Collagen deposition in the lung was quantified using NIH Image J software. Data is represented as area of collagen (blue stain) ± SEM. (C) The distance between the innermost aspect and outermost aspect of the smooth muscle was measured at 3 different positions around each airway, using NIH Image J software. Data is represented as airway smooth muscle thickness in µm ± SEM. n = 3 mice/group. An average of 10 airways was analyzed per mouse. *p<0.05, **p<0.01, # (p<0.05) represents statistically significant differences between the OVA and Alum primed mice.

Excessive IL-4/IL-13 signaling in many different cell types during pulmonary inflammation can cause airway remodeling (reviewed in [14]). Overexpression of IL-4 or IL-13 in the lungs of mice led to induction of profibrotic mediators and myofibroblast activation [6], [15]. However, the contribution of the individual receptors (Type I vs Type II R) is still unclear. Studies have shown that the Type II R is required for TGFβ production but not for fibroblast activation *in vitro* [10], [11]. To determine the role of the Type I R in airway remodeling *in vivo*, we analyzed the amount of collagen deposition and airway smooth muscle thickness in RAG2^−/−^ and γ_c_^−/−^ mice. Masson’s Trichrome staining of lung sections revealed that a small amount of collagen (shown in blue) was present around the airways in RAG2^−/−^ mice primed with alum (Figure 6A, panel a), which increased significantly upon priming with OVA (panel b). However, both alum- and OVA-primed γ_c_^−/−^ mice showed extensive collagen deposition, when compared with RAG2^−/−^ mice (panels c&d). Quantification of collagen staining using image analysis software showed that the differences were significant (Figure 6B). Furthermore, there was a marked increase in Airway Smooth Muscle thickness in mice lacking γ_c_ in comparison to their RAG2^−/−^ counterparts (panels e-h and 6C). Interestingly, there is a correlation between the degree of airway remodeling and the extent of inflammation (Figure 1) observed in the above mice. These results demonstrate that airway remodeling can occur independently of the Type I R *in vivo*. In fact, absence of γ_c_ chain enhanced collagen deposition and increased the diameter of the airway smooth muscle layer.

### Cytokine Production by Control and γ_c_ Deficient Mice

**Figure 7:**
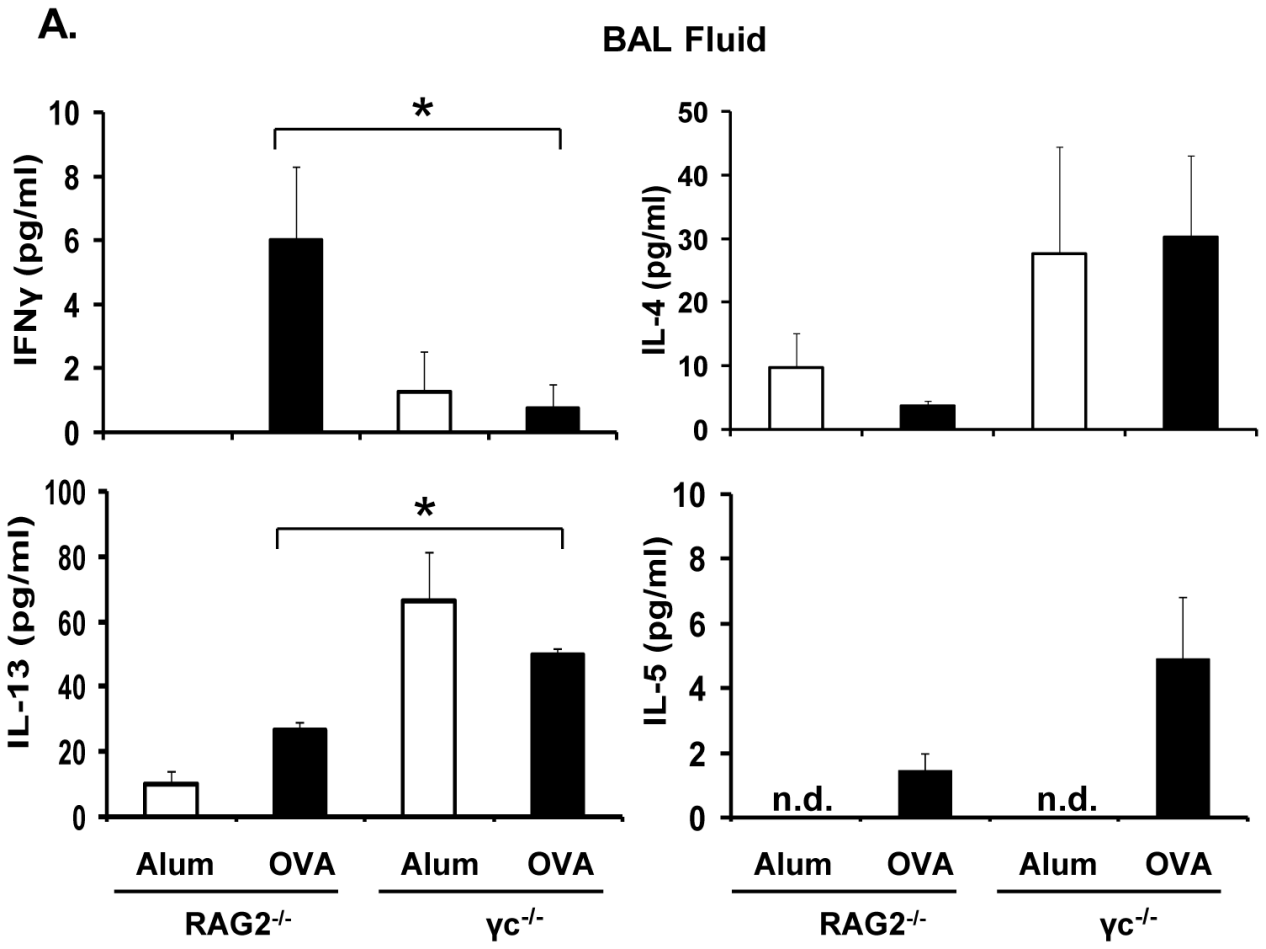
Cytokine production upon OVA priming and challenge in mice. Mice were subjected to the asthma protocol mentioned in Figure 1 and Material and Methods. Cytokine levels in BAL fluid from alum- or OVA-primed and OVA-challenged RAG2^−/−^ and γ_c_^−/−^ mice were analyzed. *p<0.05, n = 5 for OVA-primed groups, n = 3 for alum-primed. Representative data from one of two experiments is shown.

Secretion of T_H_2 cytokines generally positively correlates with the degree of inflammation. Since γ_c_ deficient mice developed severe lung pathology, we assessed the amount of IL-4, IL-5 and IL-13 present in the BAL. In the BAL, IL-13 levels were significantly greater in both alum- or OVA-primed γ_c_^−/−^ mice in comparison to RAG2^−/−^ mice; IL-4 and IL-5 levels showed a similar trend although the values did not meet the threshold for significance (Figure 7A). Along with the increase in T_H_2 cytokine levels, we observed a decrease in IFNγ secretion in the absence of γ_c_. Thus, in the absence of γ_c_ in recipient mice there was an increase in T_H_2 cytokines, even in the absence of OVA priming.

### Role of NK Cells in the γ_c_ Deficient Asthma Model

NK cell numbers are reduced in mice deficient in γ_c_ [16]. Previous studies have reported that IL-4 signaling through the Type I R induces IFNγ production by NK cells in a STAT6 dependent manner [17], [18]. Since we observed reduced levels of IFNγ in the BAL fluid of γ_c_^−/−^ mice, we tested whether reduced numbers of NK cells in these mice was responsible for enhanced T_H_2 cell activation and allergic lung inflammation. We first depleted NK cells in RAG2^−/−^ mice using antibodies against asialo GM1 using the regimen depicted in Figure S2A. If lack of IFNγ production by NK cells was causing the enhanced asthma phenotype observed in γ_c_ deficient mice, then we would expect to see the same phenotype in NK cell depleted RAG2^−/−^ mice. Treatment of RAG2^−/−^ mice with asialo GM1 antibody reduced the numbers of NK cells below the levels seen in γ_c_ deficient mice (Figure S2B). However, the degree of inflammation and eosinophilia in RAG2^−/−^ mice was still significantly lower than that observed in γ_c_ deficient mice (Figure S2C).

Next, we used an adoptive transfer approach to determine if NK cells were playing a role in this model. We adoptively transferred WT or STAT6-deficient CD49b^+^ (also known as DX5) NK cells into γ_c_xRAG2^−/−^ mice. The purity of NK cells before adoptive transfer was evaluated (Figure S3A) and the mice were then subjected to our asthma protocol (Figure S3B). We confirmed that the NK cells had repopulated into the recipient mice (Figure S3C). As observed previously, greater numbers of eosinophils were present in the BAL in γ_c_xRAG2^−/−^ mice upon OVA priming and challenge when compared to RAG2^−/−^ mice (Figure S3D). Transfer of WT or STAT6^−/−^ NK cells into γ_c_ deficient mice, however, did not change the numbers or percentages of eosinophils significantly. Thus, an alteration in NK cell numbers was likely not the reason for the enhanced allergic lung disease phenotype seen in mice deficient in γ_c_.

### Analysis of Treg and DC Populations in RAG2^−/−^ and γ_c_xRAG2^−/−^ Mice

**Figure 8:**
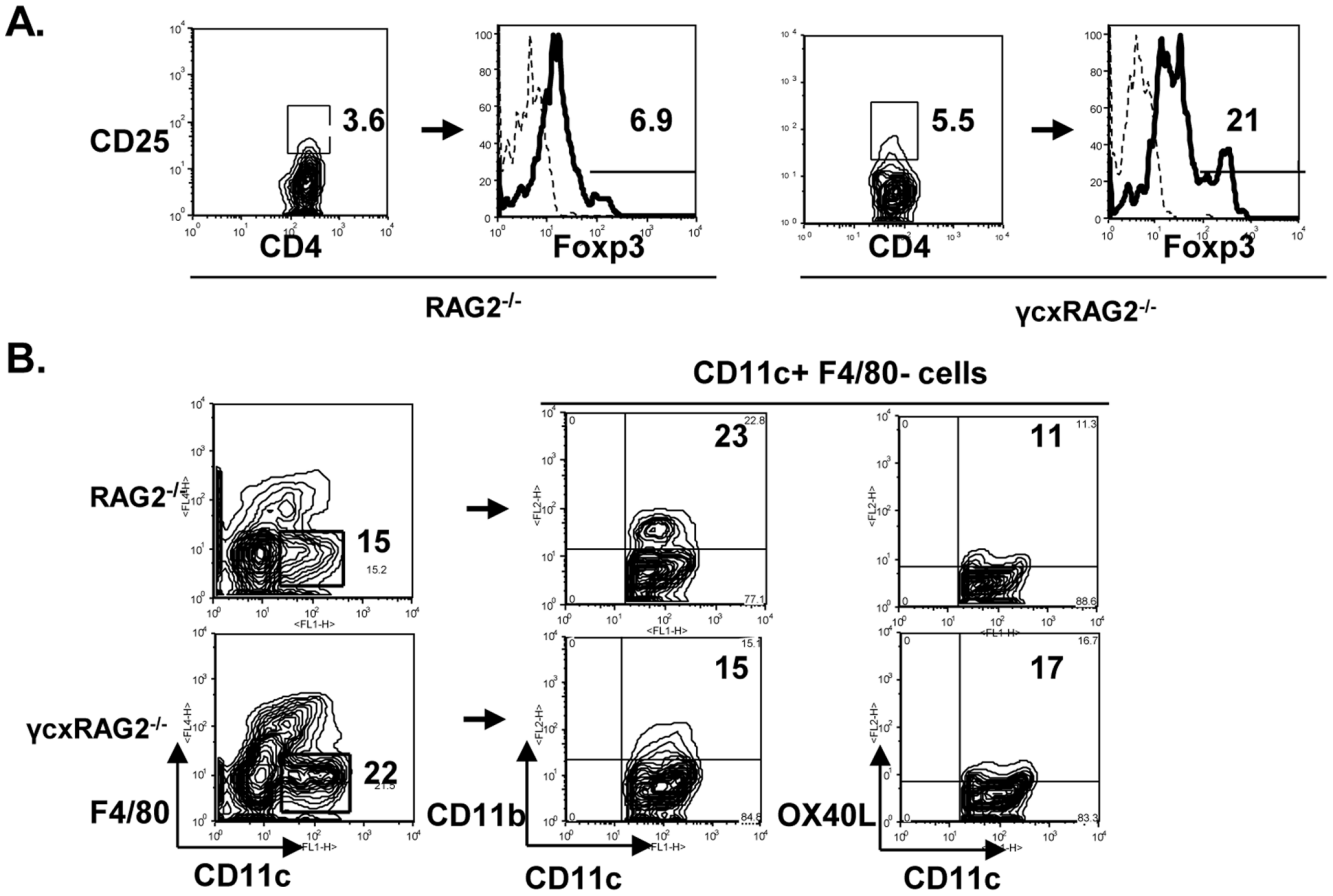
Analysis of Treg and DC subsets in RAG2^−/−^ and γ_c_xRAG2^−/−^ mice. *In vivo*-primed CD4+ T cells were adoptively transferred into RAG2^−/−^ and γ_c_xRAG2^−/−^ mice and sensitized and challenged with OVA using the protocol shown in Figure 1. Lung samples were collected 48 h after the last challenge and lung digests were performed. (A) Cells isolated from the individual lung digests (n = 3 per group) were pooled and stained with anti-CD4, anti-CD25, and anti-FoxP3. The CD4+ CD25+ cells were gated and expression of FoxP3 in these cells was studied. (B) Cells isolated from the lung digests were stained with CD11c and F4/80. The CD11c+ F4/80- cells were gated and expression of CD11b or OX40L on these cells was studied.

We transferred a population of *in vivo* primed CD4^+^ T cells that were not depleted of CD4^+^ CD25^+^ regulatory T (Treg) cells. Since regulatory T cells can suppress T_H_2 and T_H_1 cell function, we evaluated if there was a decrease in the numbers of Tregs after transfer into mice lacking γ_c_. We found, however, that the percentage of CD4^+^ CD25^+^ cells and Foxp3^+^ cells was generally low in RAG2^−/−^ mice and was a three fold increase in their numbers in the γcxRAG2^−/−^ recipient mice. Thus, there was no evidence of a reduction in Treg numbers after adoptive transfer to γcxRAG2^−/−^ recipient mice when compared to RAG2^−/−^ mice (Figure 8A).

The transferred T cells expressed γ_c_, but were placed into a γ_c_ deficient environment that could impact their subsequent responses to OVA. It is well known that dendritic cells play an important role in T cell priming. Thus, we investigated whether there was any change in the phenotype of DCs in γ_c_xRAG2^−/−^ mice. DCs in the lung were identified as CD11c^+^F4/80^−^ (Figure 8B) and CD11b and OX40L expression in these cells were monitored. While we observed an overall increase in CD11c^+^F4/80^−^ DC in γ_c_xRAG2^−/−^ recipients (22% versus 15% RAG2^−/−^), there was a reduction in the percentages of CD11b^+^ DCs in γ_c_ deficient mice from 23% to 15%. In addition, the percentage of DCs expressing OX40L was modestly increased from 11% to 17% (Figure 8B). Taken together, these results suggest the possibility that enhanced allergic lung inflammation occurring in mice deficient in γ_c_ may be caused by dysregulated activation of the adoptively transferred γ_c_^+/+^ T-cells.

## Discussion

There is overwhelming evidence linking IL-4 and IL-13 signaling to allergic asthma responses. Since IL-4 and IL-13 share receptor complexes, however, the exact contribution of the individual receptor complexes in inducing asthma pathophysiology is unclear. In this study, we used mice deficient in γ_c_ to elucidate the role of the Type I IL-4 receptor.

In addition to lacking the Type I IL-4 receptor, the γ_c_^−/−^ mice are also deficient in IL-2R, IL-7R, IL-9R, IL-15R and IL-21R. IL-2 and IL-7 signaling play an important role in lymphocyte development and survival (reviewed in [19], [20]). γ_c_^−/−^ mice lack T and B cells. Therefore, we used RAG2^−/−^ mice as controls for all our experiments. Since T_H_2 cells, and the cytokines they produce, are essential for initiation and propagation of allergic responses, we adoptively transferred *in vivo*-primed CD4^+^ T cells from OT-II (OVA specific) transgenic mice. These transferred T cells express γ_c_ and thus, receptors for IL-2, IL-4, IL-7 and IL-15. Therefore, they can respond to these cytokines even in a γ_c_ deficient environment. We have demonstrated previously that TCR transgenic mice can be immunized with OVA/alum and that transfer of *in vivo* primed CD4^+^ T cells into mice followed by OVA/alum priming and OVA challenge was sufficient to induce features of allergic lung inflammation [13].

Mice lacking the Type II IL-4/IL-13 receptor (IL-13Rα1^−/−^ mice) still developed pulmonary inflammation and eosinophlia upon allergen challenge [10], [11]. Therefore, we hypothesized that IL-4 signals through the Type I R may be uniquely responsible for inducing these effects. However, our results show that the absence of the γ_c_ chain caused no defect in these processes, suggesting that the Type I R is not absolutely required for mediating inflammatory responses and eosinophil recruitment into the lung. Since these responses are dependent on the IL-4Rα [1], the Type I and Type II receptors must mediate redundant functions for the inflammatory response.

Interestingly, we found that γ_c_ deficiency significantly enhanced lung pathology mediated by the transferred OT-II helper T-cells. While the exact mechanism involved is unknown, the increase in T_H_2 cytokines in the BAL fluid of γ_c_^−/−^ and γ_c_xRAG2^−/−^ mice, together with the reduction in IFNγ may contribute to this exaggerated asthma response. The increased amounts of IL-4 and IL-13 present in γ_c_ deficient mice could amplify signaling through the Type II R and enhance asthma responses.

We also observed that γ_c_ deficiency in mice led to enhanced airway remodeling, leading to excessive collagen deposition and increase in smooth muscle thickness. IL-13 signaling through the Type II R is considered to be the dominant inducer of fibrosis. IL-13 induces macrophages to produce TGFβ and can act directly or indirectly on fibroblasts inducing collagen and extracellular matrix deposition (reviewed in [14]). It has also been reported that both eosinophils and FIZZ1 and YM1 can cause lung fibrosis and smooth muscle thickening [21], [22], [23]. In our model, the extent of inflammation in mice correlates well with the degree of airway remodeling.

Previous studies had indicated that gene expression of AAM products in the lung were differentially regulated by the Type I and Type II receptors: YM1 mRNA expression was partially dependent on IL-13Rα1, while FIZZ1 mRNA induction was completely independent of this chain [10]. Here we show that epithelial cells in both RAG2^−/−^ and γ_c_^−/−^ mice were able to produce FIZZ1 and YM1, suggesting that induction of these proteins can occur independently of IL-4 signaling through the Type I R. We also observed that greater numbers of airways were YM1+ or FIZZ1+ in γ_c_^−/−^ mice. This was surprising, as epithelial cells usually lack γ_c_ expression. It is possible however, that the increased levels of IL-4 and IL-13 present in these mice results in greater engagement of these cytokines with the Type II R on epithelial cells, thus enhancing YM1 and FIZZ1 protein expression. Conversely, the decreased levels of IFNγ observed in the BAL fluid may be causing the enhanced T_H_2 responses seen in epithelial cells. IFNγ signaling in airway epithelial cells has been reported to suppress STAT6 activation [24].

Our group had shown earlier that IL-4 induced robust AAM gene expression in BMM cells i*n vitro* while IL-13 was less potent in inducing the same responses [12]. When macrophages lacked the γ_c_ chain and the Type I R, however, their response to IL-4 was reduced, yet IL-13 responses were intact in these cells [12]. Consistent with these *in* *vitro* studies, we found that YM1 protein expression in airway macrophages was reduced by half in the absence of the γ_c_ chain, suggesting that the Type I R regulates YM1 protein expression in macrophages *in vivo*.

The enhanced T_H_2 responses and allergic lung inflammation occurring as a result of γ_c_ deficiency was puzzling. In addition to the Type I IL-4R, the IL-9R, IL-15R and IL-21R are also absent in γ_c_^−/−^ mice. Studies in IL-9 deficient mice have demonstrated that T_H_2 differentiation, eosinophilic inflammation, AHR, mucus and IgE production occurred normally [25]; IL-9 is mainly required for mast cell function and airway remodeling in chronic asthma [26]. Importantly, these findings and other literature show a positive correlation between IL-9 and asthma pathogenesis. Thus, it is unlikely that the absence of IL-9 signaling in our model (which is mast cell-independent) is responsible for the enhanced allergic lung inflammation seen in γ_c_^−/−^ mice. IL-21 is a γ_c_-dependent cytokine that is mainly produced by activated T cells and it is required for differentiation of T_H_17 cells and T follicular helper (Tfh) cells (reviewed in [27]). Since all the mice in our model received WT T cells, however, they would be able to respond to IL-21.

IL-15 regulates NK cell development, and loss of IL-15 or IL-15R results in a profound reduction in NK cell numbers [19]. In confirmation of published studies, we found that the numbers of NK cells were reduced in mice lacking γ_c_. It has been reported that IL-4 induces IFNγ production in NK cells [17], [18] and IFNγ is known to suppress T_H_2 responses. Thus, we examined whether reduced NK cell numbers was responsible for the asthma phenotype seen in our model. We found, however, that both depletion of NK cells in RAG2^−/−^ mice or transfer of NK cells into γ_c_xRAG2^−/−^ mice did not alter features of allergic lung disease.

Recently, several groups have identified a novel population of innate lymphocytes called type 2 innate lymphoid cells (ILC2), that do not express standard lineage markers (reviewed in [28]). These cells are found in the lung and exacerbate allergic inflammation by producing IL-13 and directly inducing AHR [29]. However, they are dependent on the γ_c_ chain and are absent in γ_c_^−/−^ mice [30]. Our data suggests that in the presence of T_H_2 cells, these innate lymphocytes are not required.

It is possible that there is altered T_H_2 priming in the γ_c_ deficient environment. Since we provided a population of γ_c_^+/+^ CD4+ OT-II T cells that have been primed only once *in vivo*, we performed additional rounds of OVA/alum priming in the host after adoptive transfer. Dendritic cells play an integral role in T cell priming, and therefore, we postulated that the absence of γ_c_ on DCs may cause dysregulated T cell priming. Indeed, we observed an increase in the T_H_2 cytokine production and an increase in the percentage of Foxp3^+^ cells when T cells were primed and challenged with OVA in the γ_c_–deficient environment. It will be interesting to determine whether the Foxp3^+^ cells maintain suppressor function or acquire the ability to make effector cytokines. In addition to the T-cell changes, we found that the number of CD11b^+^CD11c^+^ DCs was reduced in γ_c_xRAG2^−/−^ mice while OX40L^+^ CD11c^+^ cells were modestly increased in these mice. DCs express both the Type I and Type II receptor and it has been established that these two receptors have differential roles in DC function. Lutz *et. al.* demonstrated that both IL-4 and IL-13 promote DC maturation by signaling mainly through the Type II R [31]. In contrast, the Type I R induces IL-12 production in DCs. It is conceivable that in γ_c_ deficient mice, absence of this negative signaling loop causes enhanced T_H_2 priming. OX40L expression in DCs is associated with increased T_H_2 differentiation in absence of IL-12 [32]. It has also been reported that the balance between myeloid DCs and plasmacytoid DCs is altered in asthma, with a significant increase in the numbers of pDCs in asthma patients [33]. The reduction in CD11b+ DCs in γ_c_xRAG2^−/−^ mice points to a reduction in the numbers of mDCs in these mice, since CD11b is a marker for this subset of cells.

In summary, these results demonstrate that expression of γ_c_ is not required for eliciting effector asthma responses such as pulmonary inflammation, recruitment of eosinophils and mucus production. In the absence of the Type I R, the Type II R is sufficient to mediate these responses. In contrast, AAM protein expression in macrophages was dependent on the Type I R. Mice deficient in γ_c_, however, developed a severe asthma phenotype when compared to control mice. Elevated T_H_2 cytokine production may be responsible for the exacerbated asthma responses seen in γ_c_ deficient mice.

## Supporting Information

Figure S1 **Activation status of** ***in vivo*** **primed CD4+ T cells from OT-II mice.** (A) Splenocytes were isolated from unimmunized or OVA/alum-immunized OT-II transgenic mice and cultured *in vitro* in media alone or with anti-CD3 and anti-CD28 for 48 hours. Cells were stained with fluorochrome-conjugated antibodies and flow cytometry was performed. The OVA-specific T cells (CD4+Vα2+Vβ5+) were gated and expression of CD44, CD62L and CD69 was monitored. (B) Splenocytes isolated from unimmunized or immunized OT-II mice were cultured in presence or absence of PMA/Ionomycin for 18 hours. ELISA was performed on cell culture supernatants.
(TIF)

Figure S2 **NK cell depletion in RAG2^−/−^ mice does not enhance allergic lung inflammation.** (A) Schematic representation of asthma protocol used in this study. Mice were primed with OVA/alum or alum alone and challenged with aerosolized OVA as mentioned in Materials and Methods. In addition, OVA/alum or alum treated RAG2^−/−^ mice were injected with anti-asialo GM1 antibodies i.p. every 5 days, starting on day -2. (B) Depletion of NK cells in RAG2^−/−^ mice were confirmed by flow cytometry. (C) Differential cell counts of BAL cells isolated from RAG2^−/−^ and γ_c_^−/−^ mice after OVA priming and challenge is depicted. *p<0.05, n = 5 for OVA-primed mice, n = 3 for alum primed.
(TIF)

Figure S3 **Transfer of NK cells does not reduce asthma responses.** (A) CD49b^+^ NK cells were enriched from the spleens of STAT6^+/+^ and STAT6^−/−^ mice as described in Materials and Methods. After enrichment, the cells were stained with antibodies to CD49b and CD3. The percentage of CD49b+ NK cells before and after enrichment is shown. (B) Schematic representation of asthma protocol used in this study. Briefly, 5×10^6^ CD4^+^ T cells were transferred into recipient mice in the presence or absence of 1×10^6^ WT or STAT6^−/−^ NK cells. Mice were primed and challenged twice with OVA on the days indicated. After the last challenge, mice were euthanized and BAL fluid and lung tissue samples were collected. (C) Spleens from recipient mice treated as described above in (B) were harvested and analyzed for expression of CD49b and CD3 by flow cytometry. (D) BAL from recipient mice treated as described above in (B) were harvested. The numbers and percentages of macrophages (Mac), eosinophils (Eos), lymphocytes (Lym) and neutrophils (PMN) present in the BAL after priming and challenge with OVA in the different groups of mice were enumerated by differential counting after cytospin. (n = 4 for each group).
(TIF)
