# Supplementary figures and images for: Absence of the Common Gamma Chain (γc), a Critical Component of the Type I IL-4 Receptor, Increases the Severity of Allergic Lung Inflammation
Source: PLoS One. 2013 Aug 5;8(8):e71344. doi: 10.1371/journal.pone.0071344 (PMC3734063; doi:10.1371/journal.pone.0071344)

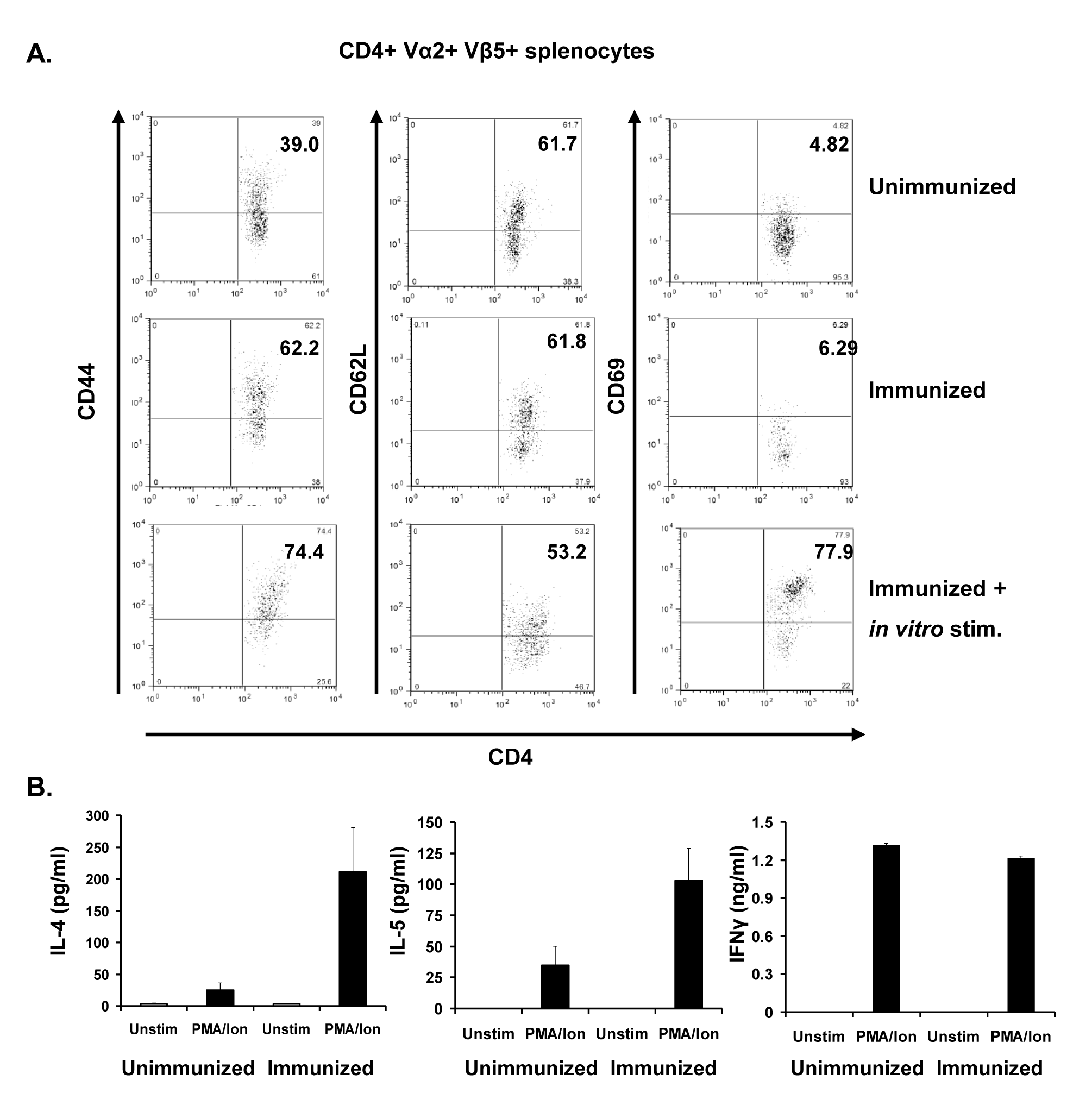

Supplement: Figure S1 — Activation status of in vivo primed CD4+ T cells from OT-II mice. (A) Splenocytes were isolated from unimmunized or OVA/alum-immunized OT-II transgenic mice and cultured in vitro in media alone or with anti-CD3 and anti-CD28 for 48 hours. Cells were stained with fluorochrome-conjugated antibodies and flow cytometry was performed. The OVA-specific T cells (CD4+Vα2+Vβ5+) were gated and expression of CD44, CD62L and CD69 was monitored. (B) Splenocytes isolated from unimmunized or immunized OT-II mice were cultured in presence or absence of PMA/Ionomycin for 18 hours. ELISA was performed on cell culture supernatants. (TIF) [file pone.0071344.s001.tif]

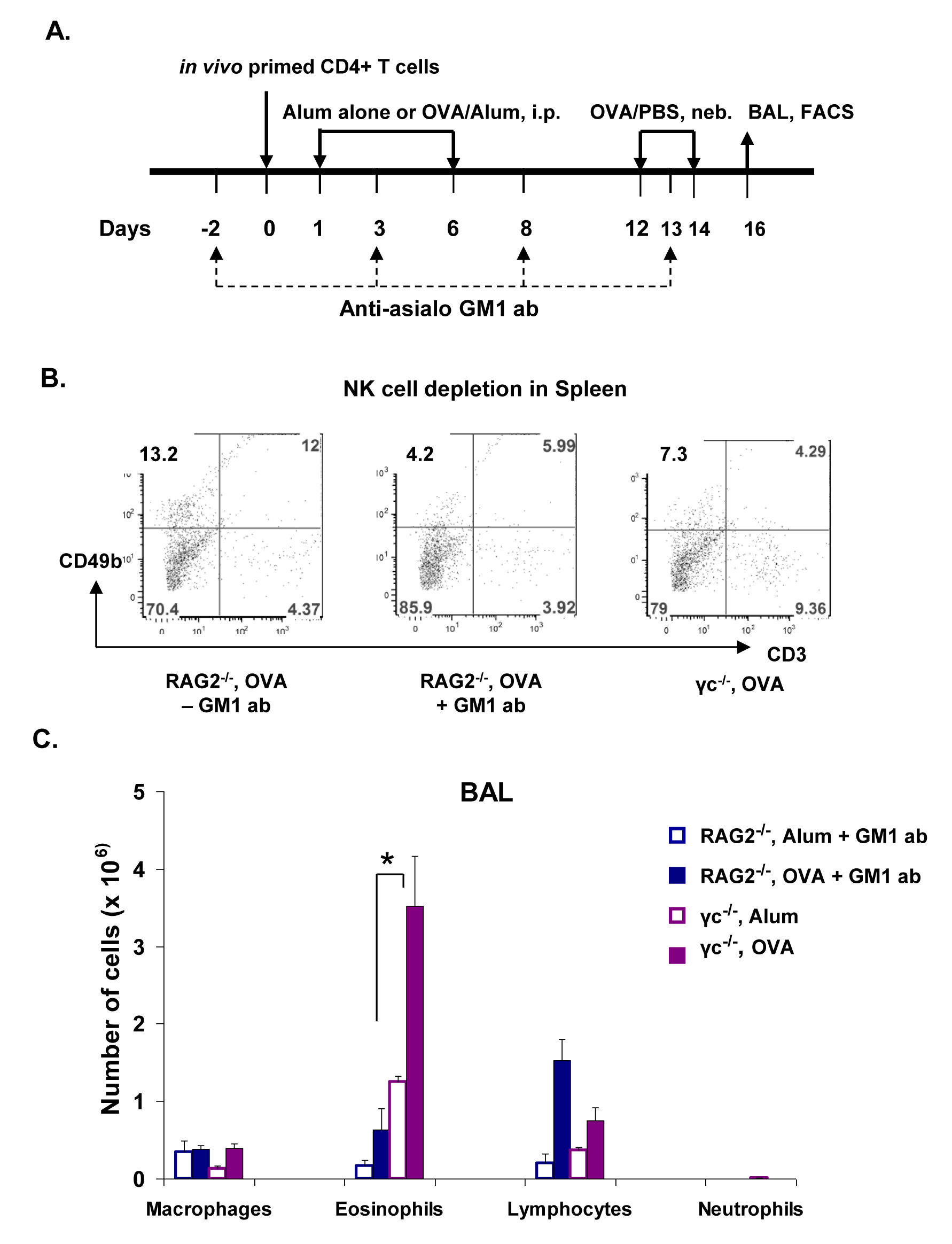

Supplement: Figure S2 — NK cell depletion in RAG2−/− mice does not enhance allergic lung inflammation. (A) Schematic representation of asthma protocol used in this study. Mice were primed with OVA/alum or alum alone and challenged with aerosolized OVA as mentioned in Materials and Methods. In addition, OVA/alum or alum treated RAG2−/− mice were injected with anti-asialo GM1 antibodies i.p. every 5 days, starting on day -2. (B) Depletion of NK cells in RAG2−/− mice were confirmed by flow cytometry. (C) Differential cell counts of BAL cells isolated from RAG2−/− and γc −/− mice after OVA priming and challenge is depicted. *p<0.05, n = 5 for OVA-primed mice, n = 3 for alum primed. (TIF) [file pone.0071344.s002.tif]

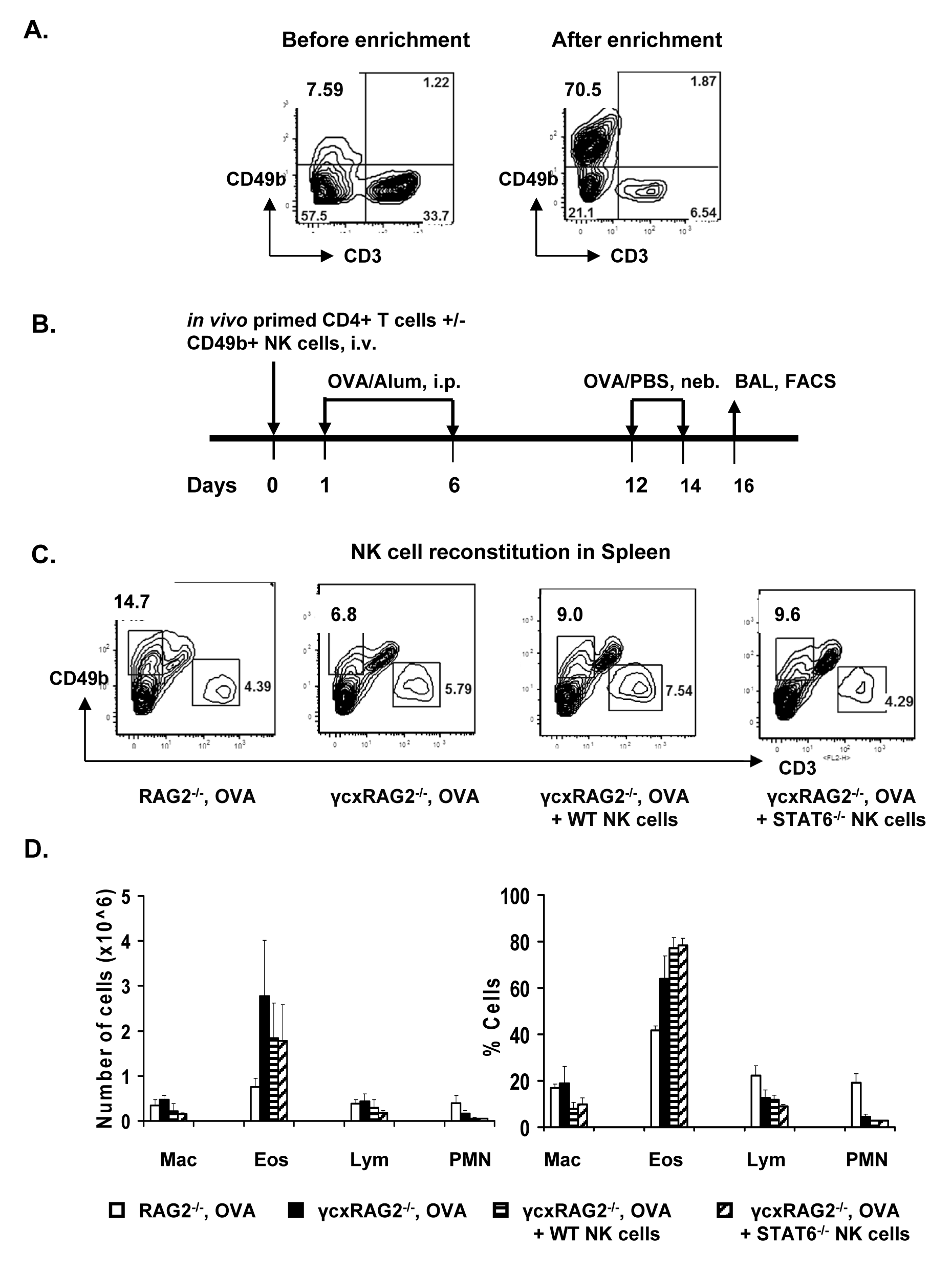

Supplement: Figure S3 — Transfer of NK cells does not reduce asthma responses. (A) CD49b+ NK cells were enriched from the spleens of STAT6+/+ and STAT6−/− mice as described in Materials and Methods. After enrichment, the cells were stained with antibodies to CD49b and CD3. The percentage of CD49b+ NK cells before and after enrichment is shown. (B) Schematic representation of asthma protocol used in this study. Briefly, 5×10^6^ CD4+ T cells were transferred into recipient mice in the presence or absence of 1×10^6^ WT or STAT6−/− NK cells. Mice were primed and challenged twice with OVA on the days indicated. After the last challenge, mice were euthanized and BAL fluid and lung tissue samples were collected. (C) Spleens from recipient mice treated as described above in (B) were harvested and analyzed for expression of CD49b and CD3 by flow cytometry. (D) BAL from recipient mice treated as described above in (B) were harvested. The numbers and percentages of macrophages (Mac), eosinophils (Eos), lymphocytes (Lym) and neutrophils (PMN) present in the BAL after priming and challenge with OVA in the different groups of mice were enumerated by differential counting after cytospin. (n = 4 for each group). (TIF) [file pone.0071344.s003.tif]
